# Self-Powered Flexible Sensors: Recent Advances, Technological Breakthroughs, and Application Prospects

**DOI:** 10.3390/s26010143

**Published:** 2025-12-25

**Authors:** Xu Wang, Jiahao Huang, Xuelei Jia, Yinlong Zhu, Shuang Xi

**Affiliations:** 1 College of Mechanical and Electronic Engineering, Nanjing Forestry University, Nanjing 210037, China; huangjiahao@njfu.edu.cn (J.H.); 2311501110@njfu.edu.cn (X.J.); shuangxi@njfu.edu.cn (S.X.); 2 State Key Laboratory of Robotics, Shenyang Institute of Automation, Chinese Academy of Sciences, Shenyang 110169, China

**Keywords:** self-powered sensors, energy harvesting, wearable electronics, advanced materials

## Abstract

Self-powered sensors, leveraging their integrated energy harvesting–signal sensing capability, effectively overcome the bottlenecks of traditional sensors, including reliance on external power resources, high maintenance costs, and challenges in large-scale distributed deployment. As a result, they have become a major research focus in fields such as flexible electronics, smart healthcare, and human–machine interaction. This paper reviews the core technical paths of six major types of self-powered sensors developed in recent years, with particular emphasis on the working principles and innovative material applications associated with frictional charge transfer and electrostatic induction, pyroelectric polarization dynamics, hydrovoltaic interfacial streaming potentials, piezoelectric constitutive behavior, battery integration mechanism, and photovoltaic effect. By comparing representative achievements in fields closely related to self-powered sensors, it summarizes breakthroughs in key performance indicators such as sensitivity, detection range, response speed, cyclic stability, self-powering methods, and energy conversion efficiency. The applications discussed herein mainly cover several critical domains, including wearable medical and health monitoring systems, intelligent robotics and human–machine interaction, biomedical and implantable devices, as well as safety and ecological supervision. Finally, the current challenges facing self-powered sensors are outlined and future development directions are proposed, providing a reference for the technological iteration and industrial application of self-powered sensors.

## 1. Introduction

Driven by the global technological trend, the deep integration of the Internet of Things, artificial intelligence, and wearable technology is reshaping the development pattern of intelligent sensors. The demands on traditional sensors have evolved from early single-parameter detection to multi-dimensional requirements of low power consumption, long battery life, and high integration [1,2]. Especially in scenarios such as distributed deployment and long-term unattended operation, the limitations of conventional sensors are becoming more evident, posing unprecedented challenges to their autonomous operation capabilities. Specifically, although piezoelectric and triboelectric sensors possess inherent self-powering potential, they are limited by their working mechanisms and can only respond to dynamic mechanical signals, failing to meet the requirements of static scenarios such as static pressure detection. Resistive and capacitive sensors rely on external power sources or grid power supply [3]. They are not only difficult to apply in remote areas, mobile devices, and other scenarios without power supply conditions, but also face problems such as high maintenance costs, frequent battery replacement, and complex wiring, which seriously restrict the construction of large-scale distributed sensor networks. Furthermore, with the enhancement of environmental awareness, the electronic waste pollution caused by the disposal of traditional sensors and the resource consumption resulting from frequent power replacement are also contrary to the concept of sustainable development.

**Figure 1:**
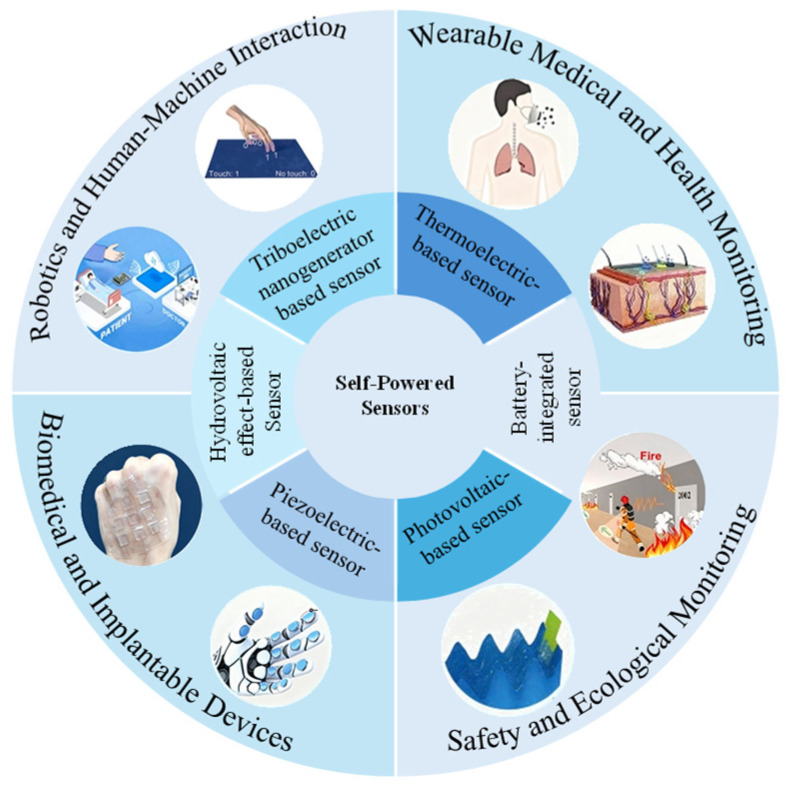
The main types and application scenarios of self-powered sensors in recent years.

Without relying on external power supply sources, self-powered sensors offer benefits such as lower weight, a more compact design, improved energy efficiency, and enhanced potential for device miniaturization. Self-powered sensors flawlessly satisfy the requirements of contemporary intelligent sensors for low power consumption, distributed deployment, and long-term monitoring by directly converting mechanical, thermal, and chemical energy into electrical energy through the cooperative design of energy harvesting and sensing units. They also achieve the perception of physical and chemical signals. The global market for self-powered sensors, which is typically applied in industrial automation [4], healthcare [5,6,7], automotive [8], smart home [9], and environmental monitoring [10], is expected to reach $34.99 billion by 2034, exhibiting an annual compound growth rate of more than 11% during the period 2025–2034 [11]. Its technological breakthroughs are of key significance for promoting the upgrade of flexible electronics and intelligent manufacturing. Moreover, ecological and environmental monitoring represent additional fields in which self-powered sensors show great application potential [12,13]. As shown in Figure 1, the main types and applications of self-powered sensors in recent years are presented.

In the fields of medical care and healthcare, this article focuses on the application of self-powered sensors in wearable medical devices and health monitoring, emphasizing their direct significance for human health and ecological security. In the area of human–computer interaction, it explores the potential of self-powered sensors in precision assembly robots, achieving multi-point touch and precise mapping through array design. In the fields of safety and ecological supervision, self-powered sensors can be used to track species population size and spatial distribution, monitor community diversity indices, and enable long-term unsupervised monitoring of ecological processes, such as carbon cycle and nutrient flows [14,15,16].

In view of typical basic mechanisms, this review systematically sorts out the core technical paths and material innovations of self-powered sensors, comparatively analyzes the performance indicators of representative achievements over the last several decades, explores their application practices in multiple fields, and looks forward to the future development trends, providing a comprehensive reference for the technological iteration and industrial application in this field.

## 2. Core Technology Pathways and Material Innovations for Self-Powered Sensors

The performance and application scenarios of self-powered sensors highly depend on their energy harvesting mechanisms. Based on the underlying energy conversion principles, self-powered sensors can be broadly classified into six mainstream mechanisms, each exhibiting distinct material requirements, operating conditions, and sensing characteristics. Broadly speaking, any device capable of harvesting energy from the environment can be regarded as self-powered. However, under a rigorous classification, RFID and NFC rely on externally induced RF energy for only instantaneous operation and cannot sustain continuous power, while Wi-Fi, Bluetooth, and cellular technologies do not inherently perform energy harvesting and strongly depend on external communication infrastructure. Such systems lack true energy autonomy and are more closely related to communication-assisted or wireless power transfer paradigms than to genuinely self-sustained sensing systems. Therefore, these electromagnetic and RF-based communication technologies are not categorized as self-powered sensors, ensuring a precise definition that focuses on sustainable energy harvesting and practical sensing applications. Although electromagnetic induction is a valid energy harvesting mechanism, its application in self-powered sensors is limited due to low output voltage at small scales, poor efficiency under low-frequency and small-amplitude excitations, bulky device structures, and mismatch with the voltage requirements of ultra-low-power electronics. Consequently, electromagnetic harvesters are more suitable for macro-scale or rotational systems rather than miniaturized self-powered sensor nodes.

This article systematically summarizes the six mechanisms of self-powered sensors and focuses on reviewing innovations in related materials and applications [17]. The following sections introduce and discuss each of these six self-powered sensing mechanisms in detail.

### 2.1. Triboelectric Nanogenerator-Based Sensor

The fundamental working principle of a triboelectric nanogenerator (TENG)-based sensor is the coupling effect of contact electrification and electrostatic induction, which enables self-powered sensing without the need for an external power source and converts mechanical stimuli (such as pressure, tactile, sliding, and vibration) into detectable electrical signals [18]. It is currently one of the most researched technologies in self-powered sensors due to its extensive material compatibility, low cost, and great sensitivity [19,20].

The differential in electronegativity between various materials is the source of triboelectric power. When mechanical contact happens to two materials with distinct electronegativities, equal-magnitude electrostatic charges of opposite signs form on their interface due to electron transfer from the lower electronegativity material to the higher one [21]. Considering the induced electron-trapping ability disparity by the existence of the energy level of distinct contacted solid surfaces, the work function and Fermi level of materials is utilized to define their triboelectric properties [22]. Relative separation, sliding, or compression of the contacted materials generate a static electric field, which drives directional free charge migration in an external circuit to produce detectable voltage or current signals [23].

The sensor structures based on TENG are primarily characterized by the triboelectric layer morphology and mechanical stimulus transmission [24,25]. In 2010, based on the topography of the triboelectric layer, a vertically integrated ZnO nanogenerator was used to power the nanowire pH sensor and the nanowire UV sensor [26], demonstrating a self-powered system completely composed of nanowires. The TENG sensor based on nanostructures can directly convert the accelerated changes in the vascular wall into electrical signals, eliminating the differential operations and post-processing errors in traditional sensors. Since the second-order derivative waveforms are more sensitive to early-stage arteriosclerosis and arrhythmias, they can also amplify pathological features [27]. Similarly, when leaf microstructure is introduced, the sensing capacity can also be enhanced [28]. Generally speaking, the regulation of interfaces and morphologies of sensing materials are key to sensing qualities [29].

The process of signal generation in different structures emphasizes different aspects. The following are the frequently mentioned structures:

(1) Solid–solid contact separation-type TENG sensor

**Figure 2:**
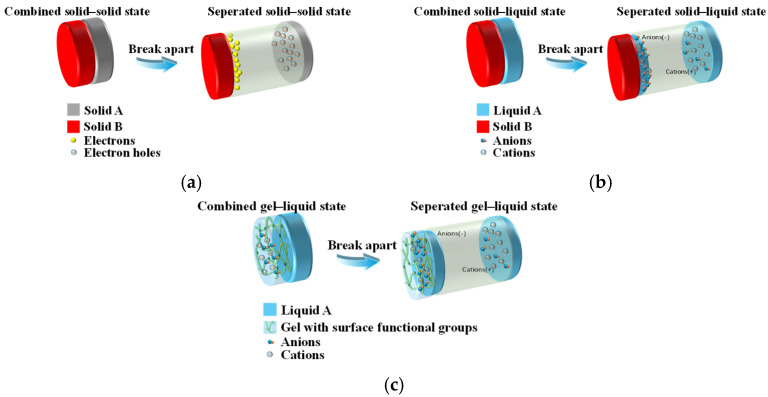
The working principles of different types of TENGs. (**a**) Working mechanism of solid–solid contact separation-type TENG; (**b**) Schematic diagram of the solid–liquid contact separation-type TENG sensor principle; (**c**) Working mechanism of gel-based TENG.

As seen in Figure 2a, the most traditional sort of sensor is the working mechanism of solid–solid contact separation-type TENG [30,31,32].

The surface microstructures of the triboelectric layer—such as micro-cone, porous textures, crumpled films, and nanowire arrays—are designed to enhance charge-transfer efficiency and sensing performance by increasing both the effective contact area and its rate of variation under mechanical stimulation [33].

(2) TENG sensor of the solid–liquid contact separation type

As seen in Figure 2b, this type of sensor overcomes the wear restriction of conventional solid–solid friction and uses the material (such as water or electrolyte) as a sort of triboelectric layer [34]. The contact separation or flow of the liquid with the solid surface is how the TENG sensor detects the transfer of charge [35].

(3) TENG sensor based on gel

Hydrogels and organic gels are widely employed as triboelectric layers or electrode materials in gel-based TENG sensors, offering a balance of biocompatibility, ionic/electronic conductivity, and mechanical flexibility, making them well suited for wearable applications [36,37]. The underlying sensing mechanism arises from the gel’s ionic conductivity coupled with the triboelectric effect [38,39], as illustrated in Figure 2c.

For TENG sensors, the macroscopic dielectric electrification behavior—such as charge density and polarity—is closely linked to the electron-donating or electron-withdrawing surface functional groups within the polymer’s repeat units. These characteristics can be described through parameters such as work function, interfacial barrier, electron affinities, energy-band structure, crystallinity, and defect states [40].

Flexible electrode materials must strike a compromise between conductivity and flexibility; the most popular options are conductive hydrogels, silver nanowires (AgNWs), and MXene (Ti_3_C_2_T_x_). Wood-based electrodes, with their naturally porous structure, biodegradability, and flexibility after chemical treatment, have achieved a good balance between electrical conductivity and mechanical compliance, demonstrating great potential as sustainable self-powered sensing materials [41,42,43,44,45,46].

### 2.2. Thermoelectric-Based Sensor

**Figure 3:**
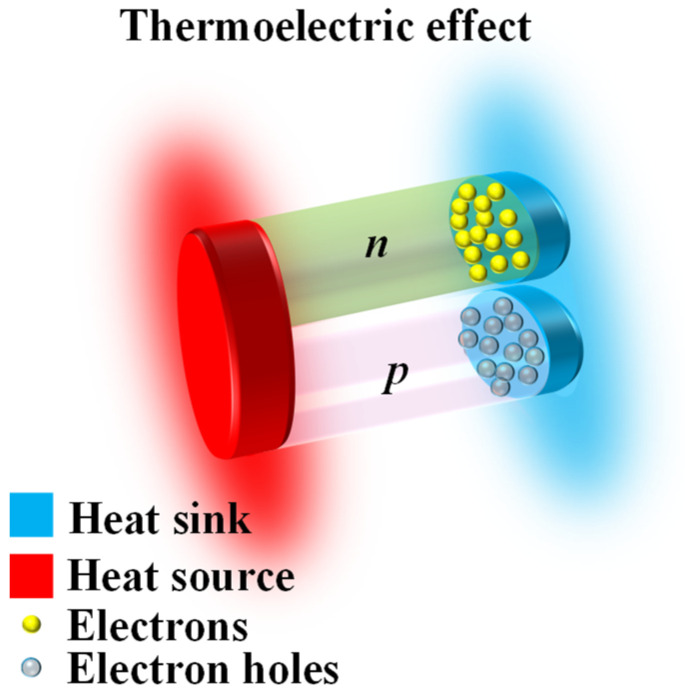
Working mechanism of thermoelectric-based sensor.

Thermoelectric sensors are based on the Seebeck effect [47] illustrated in Figure 3, where a temperature difference drives charge separation, converting the temperature gradient into a thermoelectric potential within the thermoelectric material. By utilizing the quantitative relationship between temperature and thermoelectric potential, high-precision temperature sensing can be achieved [48]. These devices combine mechanical flexibility, detection sensitivity, and self-powering capabilities, making them widely applicable in various scenarios such as industrial environment monitoring and wearable health detection [49,50].

### 2.3. Hydrovoltaic Effect-Based Sensor

By leveraging the hydrovoltaic effect, ambient humidity and aqueous solutions, and functional materials that enable ion migration or interfacial charge separation, it is possible to convert the potential or chemical energy of water into electrical energy [51,52,53,54]. Unlike piezoelectric or triboelectric nanogenerator (TENG) devices, which rely on structural displacement variations to produce electricity, hydrovoltaic sensors can operate under continuous pressure at a fixed position [55]. Among the available materials, micro-biofilms stand out for their abundance, reusability, and environmental friendliness [56]. Their primary categories are as follows:

(1) The Ion Migration Effect Driven by Humidity Gradient

**Figure 4:**
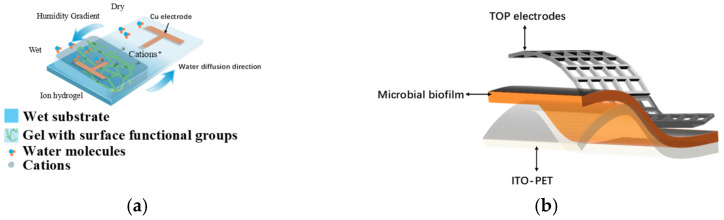
(**a**) Working principle of hydrovoltaic pressure sensors; (**b**) structure of microbial biofilm-based hydrovoltaic pressure sensors.

The functional layer of a hydrovoltaic sensor is typically composed of materials such as hydrophilic polymers or microbial biofilms [57]. The working principle of hydrovoltaic pressure sensors is shown in Figure 4a. As a new type of hydrovoltaic functional material, microbial biofilms combine ion transport channels [58] and natural porous structures [59]. Humidity gradients can drive directional ion migration on the surface of the biofilm, thereby generating continuous potential and current. For example, in the biofilm of *Geobacter sulfurreducens*, the membrane contains abundant hydroxyl groups (-OH), carboxyl groups (-COOH), and other hydrophilic functional groups that can adsorb and convert water molecules from the atmosphere into ions [38,60,61,62]. When a humidity gradient is formed across the membrane, the ions migrate along the gradient and produce a stable hydrovoltaic voltage at the electrodes (with an open-circuit voltage reaching up to 0.45 V), providing a stable power source for the sensor [63]. According to experimental results of this study, under the condition of applying pressure every 25 s, the sensor’s accuracy loss is about 3%, allowing high-precision output. However, when the ambient relative humidity drops below approximately 50%, the response current rapidly decreases, indicating that the stable operation of the hydrovoltaic sensor relies on a relatively humid environment.

How porous structures reduce ion migration energy barriers in microbial biofilms needs to be explained. The theoretical model was proposed as follows [64]:

(1) $$G=\frac{\sigma g}{\pi L}\ln\left(\frac{4L}{a}\right)$$

where *G* is the conductance, σ refers to pili conductivity, g is biofilm thickness, L is the electrode length, and a is the pore radius. Porous networks could create direct tunneling paths between hydration layers, bypassing insulating organic matrices, and amplify local electric fields via nanoscale charge accumulation. As shown in Figure 4b, it is the structure of microbial biofilm-based hydrovoltaic pressure sensor.

(2) Solid–liquid interface charge separation effect

At solid–liquid interfaces, where hydrophobic PDMS, microbial nanowires are frequently adopted as the solid surface and water or aqueous solutions as the liquid, the charge separation effect adsorbed ions of H^+^ from the dissociation of water molecules on the solid surface form an electric double layer, and when solid materials come into contact with liquids, charge transfer takes place at the solid–liquid interface because of the difference in electron affinity between the material and water molecules. The electric double layer’s equilibrium is upset when an external pressure modifies the solid–liquid contact or the ion migration path, which results in a redistribution of charge and observable changes in electrical signals.

The core working principle of potential-type self-powered sensors is the coupling of energy conversion driven by ion gradients and stimulus response. The ion gradient is constructed through the design of the device structure, and the ion gradient is used as the self-powered energy source. The ion gradient promotes the directional migration of ions such as H^+^ and OH^−^, forming a double electric layer (EDL) with a thickness of only the nanometer level at the interface between the electrode and the ionic conductive layer, generating a stable open-circuit potential difference. When external stimuli are applied, the ion flux and EDL capacitance can be regulated by changing the ion migration path, the contact area between the electrode and the ion layer, or the density of the ion channel, thereby converting the physical stimuli into detectable electrical signals (voltage/current changes), achieving continuous monitoring of static and dynamic signals [65,66,67,68]. This article mainly discusses the following two types of potentiometric sensors:

(3) Ion gradient pressure sensor

The ion gradient pressure sensor establishes the potential difference by constructing a gradient ion distribution, and the ion conductive layer integrates a gradient microstructure. When pressure is applied, the microstructure undergoes compression deformation, reducing the interfacial air gap, increasing the contact area between the electrode and the ion layer, simultaneously densifying the ion channels, lowering the resistance to ion migration, promoting rapid ion migration, and resulting in a significant change in the EDL capacitance. The greater the pressure, the more obvious the expansion of the contact area and the optimization of the ion channel, and the stronger the electrical signal response. Eventually, the pressure magnitude is quantified through changes in capacitance or current. Some designs can maintain high linearity and high resolution within a wide pressure range (0.08 Pa–360 kPa) [69,70].

In terms of ion-conducting layer materials, laser-induced gradient micro-pyramid ion-conducting gels have been developed at present, and the sensitivity and linear range are balanced through programmable microstructure design. The staged fillable structure PVA/H_3_PO_4_ gel is adopted, and the deformed structure is accommodated by indentations and grooves to enhance compressibility. High-porosity PU/PDMS foam impregnated with ionic liquid reduces modulus and enhances ion migration efficiency, with a sensitivity of up to 9280 kPa^−1^.

(4) Ion gradient strain sensor

The ion gradient strain sensor uses an elastic substrate as the carrier to construct an axial or planar ion gradient. When subjected to tensile, bending, and other strains, the deformation of the elastic substrate will lead to the extension of the ion channels in the ion-conducting layer, the reduction in the cross-sectional area, or the expansion of the electrode spacing, increasing the resistance to ion migration. At the same time, it will change the spatial distribution of the ion gradient, causing regular changes in the EDL capacitance or device resistance. During strain recovery, the ion channels and gradient distribution return to their initial states, and the electrical signals recover synchronously, thereby achieving quantitative strain detection. The detection range can cover 0.5% to 100% strain [71,72].

In terms of elastic base and ion-conducting layer materials, stretchable elastomers (TPU, Ecoflex) loaded with ionic liquids are currently being developed to form ion-conducting gel fibers, which possess both high stretchability (>100%) and ionic conductivity. PAAm/NaCl hydrogel composite CNTs enhance the deformation recovery rate through hydrogen bond networks and are suitable for human motion monitoring [71,72].

These sensors’ primary advantages are their self-powering nature, extremely high sensitivity, and environmental compatibility, which provide a novel approach to environmental, skin, and wearable health monitoring [65].

Ionic hydrogels are the core component of ion-migration-type hydrovoltaic sensors, and their performance can be significantly enhanced by adjusting the network structure and ion concentration [66]. Semi-interpenetrating multi-ionic liquid network hydrogels incorporating LiBr as an antifreeze agent enable stable sensors across a wide temperature range from −78.5 to 60 °C, while detecting pressures from 0.4 to 25 kPa through a “moisture gradient-ion migration” coupling mechanism [63]. MXene pressure sensors based on ionic diode mechanism can achieve a sensitivity of 0.013 V/kPa and a humidity response of up to 0.93 mV/RH% [67,68].

By utilizing the abundant hydrophilic functional groups within the *G. sulfurreducens* biofilm, internal resistance changes caused by membrane compression can be monitored to enable static pressure detection. This system demonstrates a high sensitivity of 8968.7 kPa^−1^ and a rapid response time of 112.5 μs and is capable of continuous power generation for 80 h at 92% relative humidity [63]. Moreover, the stable formation of a moisture gradient provides valuable insights for the development of implantable biosensors.

### 2.4. Piezoelectric-Based Sensor

Organic piezoelectric materials, also known as piezoelectric polymers, include PVDF, nylon, PVC, polypropylene (PP), and so on. Due to their low acoustic and mechanical impedance, they are considered one of the environmentally friendly alternatives to traditional inorganic PZT [68,73,74]. Their piezoelectric performance can be further enhanced through doping copolymer modification. For example, materials such as BaTiO_3_ -doped PVDF-TrFE have shown significant enhancement in piezoelectric response [71,72]. Taking MXene-doped PVDF-TrFE as an example, its piezoelectric coefficient *d*_33_ increased from 25 pC/N to 42 pC/N. Piezoelectric sensors made from this composite material exhibit a sensitivity of 25.6 pC/N in the operating range of 0.1–50 kPa, and a response time of about 20 ms, which can meet the requirements for monitoring dynamic bending of human joints such as elbows. Moreover, compared with TENG-based sensors, these piezoelectric sensors not only perform similarly in high-frequency and wide-band monitoring but also offer advantages such as simpler fabrication process, longer service life, and higher sensing capacity [70].

A novel piezoelectric composite material—BaTiO_3_/polyacrylonitrile (PAN) elastomeric composite—significantly enhances overall performance through a synergistic effect [70]. This material exhibits high sensitivity of 3.2 kPa^−1^ within a pressure range of 1–10 kPa and maintains excellent stability over more than 10,000 cycles. The performance enhancement is mainly attributed to the improved interfacial polarization effect. Additionally, this composite has been successfully applied in tactile feedback systems of smart prosthetics, demonstrating promising practical potential [75].

The piezoelectric constitutive relationship forms the theoretical basis of piezoelectric sensors, which detect mechanical parameters such as pressure, strain, and vibration by the polarization changes induced by the deformation of piezoelectric materials under external forces [76]. By integrating nanofiber scaffolds or thin-film composite materials, sensor structures can be designed flexibly. Through doping or constructing hierarchical pore structures, various functional modifications can be achieved. As a result, self-powered sensors based on the piezoelectric effect can be realized and adapted to a variety of application scenarios [77,78,79,80]. Their fast response speed and compact structure with a thickness of less than 100 μm make piezoelectric technology highly valuable in fields related to dynamic signal measurement [69,81]. The ultrasonic field generated by piezoelectric devices can also be utilized to enhance the lower detection limit of gas sensors [82,83,84].

### 2.5. Battery-Integrated Sensor

Battery-integrated sensors utilize the electrochemical properties of batteries to sense physical signals such as pressure and stretching, achieving a fusion of energy storage/conversion and sensing functions [85]. The core mechanism involves using mechanical stimuli to regulate charge migration or impedance characteristics within the battery, thereby converting mechanical signals into recognizable electrical signals [86,87]. Devices of this type are self-powered and do not require an external power source, making them particularly suitable for low-power applications such as wearable technology and the Internet of Things.

**Figure 5:**
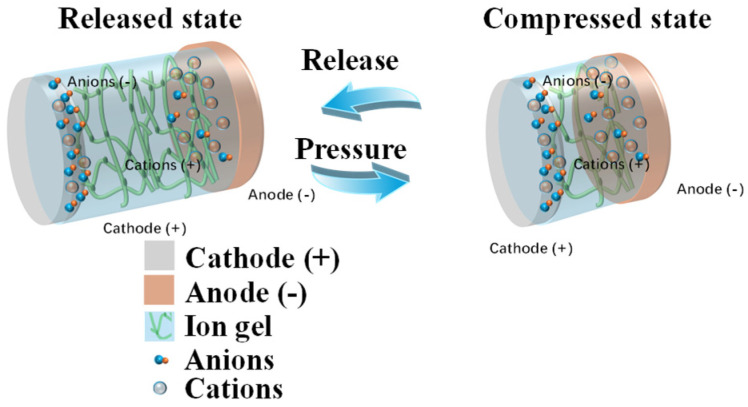
The principle diagram of the response mechanism of the gelatin battery pressure sensor.

The design and working principle of a gelatin-based battery pressure sensor are shown in Figure 5. Typically, metal cations are released from the battery anode and migrate to the cathode through the ion gel, where redox reactions occur at both the cathode and the anode, generating a voltage difference. The reaction equations for the cathode and anode are shown in Equations (2) and (3), respectively.

Cathode (+): αMetal cations (+) + Oxide + γe− ⇌ Metal compound(2)

Anode (−): Metal atom ⇌ Metal cation (+) + βe−(3)

In fact, artificial batteries still have room for improvement in terms of energy conversion efficiency and can continue to draw inspiration from biological systems in nature [88]. Genomic research has shown that the muscle cells of electric eels have evolved a unique subtype of voltage-gated sodium channels through gene duplication, specifically for generating electricity, enabling them to discharge voltages exceeding 600 V [89]. Volta’s invention of the battery was also inspired by the anatomy of electric eel [90].

Due to their extremely high safety, cost effectiveness, and environmental friendliness, zinc-ion batteries are considered the most promising integrated solution. A hydrogel zinc-ion battery sensor was developed using a PDMS isolation layer and a gelatin-chitosan composite film that serves as both the electrolyte and sensing layer. This sensor can operate in an open-circuit state without any applied pressure, and when pressure is applied, the resistance of the isolation layer decreases, enabling a pressure detection range of 0–524 kPa with a sensitivity of 37.9 mV/kPa [87].

### 2.6. Photovoltaic-Based Sensors

The workflow of photovoltaic self-powered sensors can be divided into four key stages as follows: energy harvesting, energy storage, energy management, and sensing execution, forming a complete self-powered closed-loop system. As the core of energy input, photovoltaic modules convert light energy into electrical energy through the photovoltaic effect, and their spectral response needs to be matched according to the specific application scenarios. Affected by day-night changes and fluctuations in light intensity, the system usually requires an energy storage module—such as supercapacitors [91] or flexible batteries [92]—to ensure a continuous power supply.

Dynamic energy management relies on low-power microcontrollers and related algorithms, with core technologies including maximum power tracking [93] and dynamic load regulation [94]. With energy support, the sensing module completes signal acquisition and wireless transmission, and its performance optimization mainly lies in using low-power sensing devices [95] and appropriate wireless communication protocols [91].

Material innovations in self-powered photovoltaic sensors focus on improving energy conversion efficiency, enhancing device flexibility and safety, and meeting the demand for further miniaturization. The technological pathway primarily encompasses the following three key aspects: photovoltaic power generation, energy storage, and system integration. By combining XY1 and L1 dyes, and leveraging their complementary molecular size characteristics, a denser and full coverage can be achieved on the TiO_2_ surface, effectively passivating electron recombination sites and increasing the device’s open-circuit voltage (V_o_c) to 910–1000 mV. Under 1000 lux, the device’s power conversion efficiency (PCE) can reach 34–38%, representing an improvement of over 40% compared to a single dye system. Additionally, compared with traditional cobalt-based electrolytes, the Cu^2+^/Cu^+^ (tmby)_2_ electrolyte exhibits approximately twice the electron regeneration rate, low toxicity, and only one-third of the cost, making it more suitable for large-scale applications [91,94].

The heterojunction formed by the two-dimensional material PdSe_2_ and a silicon nanowire array achieves broadband photoresponse from ultraviolet to mid-infrared, thanks to PdSe_2_’s wide-spectrum absorption (covering 265 nm to 4.6 μm) and a carrier mobility of up to 1000 cm^2^/(V·s). The device’s dark current can be effectively reduced to 10^−10^ A, and the detection rate in self-powered mode can reach 10^6^ Jones. It is also compatible with multispectral sensing applications, including night imaging and gas detection [96].

### 2.7. Compatibility Relationship Between Materials and Sensor Types

Due to significant differences in energy conversion working principles and application scenarios, self-powered sensors place highly differentiated demands on material performance. Rather than pursuing universal material solutions, effective sensor design requires a close match between functional materials and the specific working principles involved.

In triboelectric nanogenerators (TENGs), high-molecular-weight polymers such as polydimethylsiloxane (PDMS), fluorinated ethylene propylene (FEP), and polytetrafluoroethylene (PTFE) are commonly employed as friction layer materials [41,42,43]. These materials offer favorable charge affinity while maintaining mechanical compliance. However, long-term performance degradation caused by solid-solid friction remains a persistent challenge. To enhance wear resistance, a lotus-leaf-inspired superhydrophobic structure was developed and it uses water as the separation medium. By incorporating PTFE into PDMS, PTFE’s high electronegativity was used to improve charge-transfer efficiency. Meanwhile, the biomimetic lotus-leaf superhydrophobic surface enables rapid and stable solid–liquid contact separation, effectively mitigating the wear issues inherent in traditional solid–solid friction systems [34].

In contrast, thermoelectric devices rely on fundamentally different material characteristics dictated by the Seebeck effect. High Seebeck coefficients, low thermal conductivity, and thermal stability are essential for efficient thermoelectric conversion. Among various candidates, bismuth telluride (Bi_2_Te_3_) and antimony telluride (Sb_2_Te_3_) are the most commonly used core thermoelectric materials, with the Seebeck coefficient of about 3.77 mV/K at room temperature, demonstrating excellent thermoelectric conversion capability. When integrated into flexible systems, polyimide (PI) substrates are commonly used due to their ability to support thin-film structures while providing good mechanical robustness under repeated deformation [97,98]. Thermoelectric films composed of n-Bi_2_Te_3_/p-Sb_2_Te_3_, deposited onto flexible polyimide (PI) substrates using magnetron sputtering, demonstrate excellent performance [99]. The Sb_2_Te_3_ layer reaches a power factor of 18.6 μW·cm^−1^·K^−2^, and by optimizing the deposition temperature to about 35 °C and maintaining a working pressure of 2 Pa, the films exhibit stable thermoelectric behavior over a broad temperature range from −20 to 80 °C [47,48,49].

Biological sensory systems provide a compelling reference for the development of self-powered sensing technologies. In natural organisms, sensory functionality is closely coupled with continuous energy acquisition [100]. Plants such as the Venus flytrap and Mimosa pudica not only rely on photosynthesis for metabolic energy but also exhibit rapid mechanical responses to external stimuli, allowing them to perform adaptive actions such as prey capture or defensive motion [101,102]. These examples illustrate how sustained energy harvesting can support persistent and responsive sensing behavior, offering valuable design insights for next-generation electronic skins [103].

From an energetic perspective, maintaining continuous sensing functions requires a non-negligible energy supply. For instance, an adult weighing 70 kg with a body fat percentage of about 20% consumes approximately 7000 KJ (about 1650 kcal) of energy per day at the basal metabolic level. This comparison underscores the importance of efficient and sustainable energy utilization in the design of long-term self-powered sensing systems. The energy required for daily life activities mainly comes from the energy stored in adipose tissue, which can be steadily and gradually converted into adenosine triphosphate (ATP) and heat to maintain body functions [104]. In contrast, the energy density of current commercial lithium-ion batteries is about 250–300 Wh/kg. Based on this estimate, a modern lithium-ion battery weighing about 10 kg could meet the energy needs of a human at the level of basal metabolism [105].

Various energy sources related to the human body—including temperature fluctuations, heat dissipation, blood pressure changes, sweating, and various bodily movements (such as walking, swinging, and joint rotation)—can be relatively easily harvested and converted into electrical energy. The approximate power that can be harvested from different physiological activities of the human body is as follows: body heat dissipation 2.4–4.8 W, exhalation about 1.0 W, blood pressure pulsation about 0.93 W, respiration-related movement about 0.83 W, arm swinging about 60 W, finger movement about 6.9–19 mW, and walking about 67 W [106]. In addition, magnetoelastic generators (MEGs), as an emerging self-powered sensing technology, are showing broad development prospects [107,108,109].

## 3. Characteristics and Performance Evaluation of Self-Powered Sensors

Based on material and structural innovation, self-powered sensors have achieved significant breakthroughs in core indicators such as sensitivity, detection range, response time, cycle stability, and self-powered mode and efficiency. The following introduces representative achievements in these core indicators around the world in recent years:

### 3.1. Sensitivity and Detection Range

**Figure 6:**
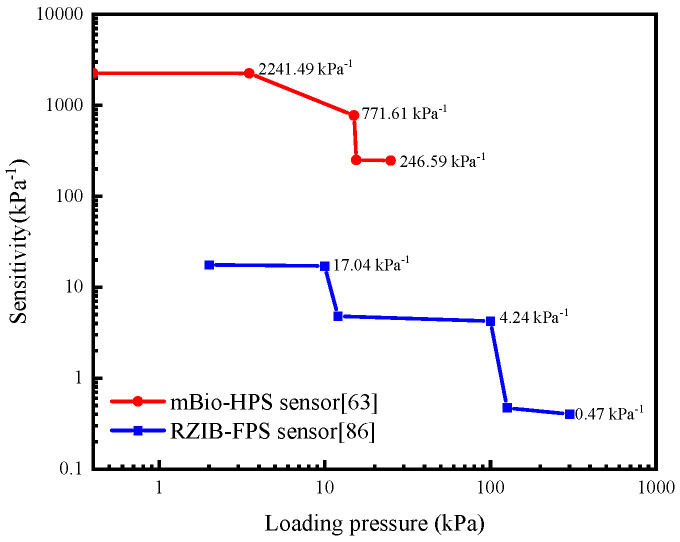
Comparison of pressure sensitivities of representative self-powered pressure sensors reported in the literature. The sensitivity values are extracted from Refs. [63,86], in which the red curve shows the mBio-HPS current response varies with pressure [63], while the blue curve shows the RZIB-FPS sensor sensitivity to external pressure [86].

Hu et al. [63] discovered an innovative microbial biofilm-based hydrovoltaic pressure sensor (mBio-HPS), which can detect particle-level pressure as low as 16 Pa. As shown by the red curve in Figure 6, the sensitivity in the low-pressure region of 0.4–3.5 kPa is 2241.49 kPa^−1^, indicating its special ability to detect weak forces. The sensitivity in the high-pressure area of 15.5–25 kPa is 246.59 kPa^−1^. Hu et al. [86] reported the design and fabrication of a new rechargeable Zn-ion battery-type flexible self-powered pressure sensor (RZIB-FPS). The sensitivity of the zinc-ion battery sensor is 42.6 mV/kPa in the low-pressure region below 10 kPa, 10.6 mV/kPa in the 10–126 kPa range, and 1.18 mV/kPa in the 126–330 kPa range, which could be normalized by a low background voltage of about 2.5 mV, as illustrated by the blue curve in Figure 6 [86].

**Figure 7:**
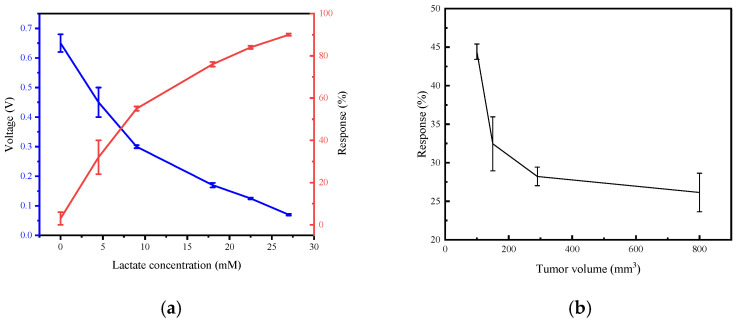
Sensing characteristics of a self-powered lactate sensor [77]. (**a**) The response of the self-powered sensor in different lactate solutions; (**b**) the response of the device for lactate sensing of tumors (Adapted from Ref. [77] under CC BY 4.0 license. https://doi.org/10.3390/s24072161).

Wang et al. [110] reported a self-powered flexible temperature-pressure bimodal sensor based on high-performance thermoelectric films and porous microconed conductive elastic materials. Under a stable cold stimulus of 31 °C with a temperature difference of 2 °C, relative to 33 °C, the temperature sensor generated a periodic voltage output of approximately 8.1 mV during repeated loading-unloading cycles. In addition, a comprehensive comparison of the performance of different types of thermoelectric sensors under different test conditions is presented in Table 1 [99]. Beyond physical sensing, self-powered sensors have also been extended to biochemical monitoring applications. As shown in Figure 7a, the self-powered lactate sensor based on lactate oxidase-modified ZnO nanowire arrays show high sensitivity with a low detection limit of approximately 1.3 mM, a broad detection range up to at least 27 mM [77]. Figure 7b further illustrates that the sensor response varies with tumor size, indicating its potential for biomedical monitoring.

Self-powered sensors can signals spanning a wide range, from macro-scale voltages up to 10 kV [110] and stresses of 100 MPa [111] to micro- and nano-scale signals such as nC-level charges [112] and pressures of only several Pa [9]. To better evaluate the sensing capabilities and practical performance, the benchmark performance of thermoelectric and piezoelectric sensors is compared in Table 2.

**Figure 8:**
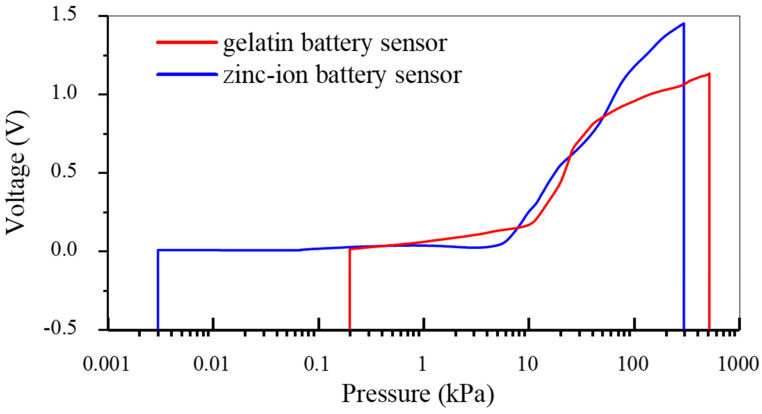
Pressure-voltage response curves of different battery-based pressure sensors under external mechanical pressure reported in the literature. The sensitivity values are extracted from Refs. [86,87], in which the blue curve shows the voltage response of the zinc-ion battery pressure sensor to applied pressure [86], while the red curve shows the voltage response of the gelatin battery pressure sensor to applied pressure [87].

Jia et al. [87] reported a gelatin-based battery-type pressure sensor. As shown by the red curve in Figure 8, the gelatin battery sensor has a pressure detection range of 200 Pa to 524 kPa. It can detect the P, T, and D waves of wrist pulse waves under a micro-pressure of 200 Pa and is suitable for industrial small-load detection under a high pressure of 524 kPa. Hu et al. [86] reported the design and fabrication of a new rechargeable Zn-ion battery-type flexible self-powered pressure sensor (RZIB-FPS). As shown by the blue curve in Figure 8, the zinc-ion battery sensor has a pressure detection range of 3 Pa to 330 kPa. It can sense the impact of a single water droplet at 3 Pa and can be adjusted to monitor heavy-duty movements such as deep squats at 330 kPa [63], with a response time of 112.5 μs and a recovery time of 130.3 μs.

**Figure 9:**
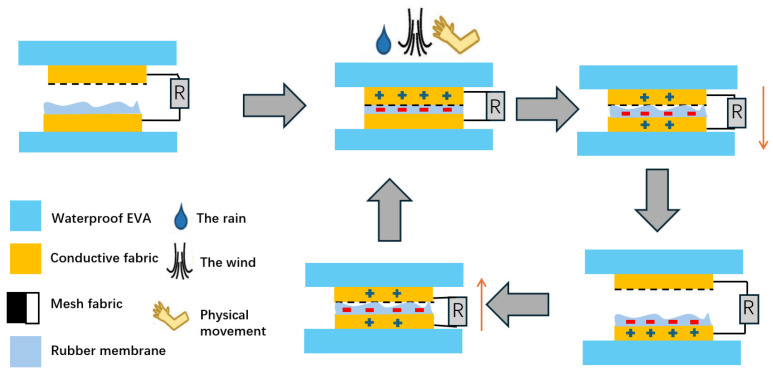
The working principle of WPF-MTENG (Adapted from Ref. [33] under CC BY 4.0 license. (https://doi.org/10.1002/advs.201801883).

Lai et al. [33] presented the first single waterproof and fabric-based multifunctional triboelectric nanogenerator (WPF-MTENG). The working principle of this sensor is shown in Figure 9, mainly involving contact friction charging and electrostatic induction. TENG based on waterproof fabrics can harvest energy from various sources, including raindrops, wind, and human motion. This enables it to indirectly cover a pressure range of 0.1 N to 10 N, making it suitable for wearable and outdoor environments. Wang et al. [71] reported a piezoelectric film pressure sensor fabricated by electrospun polyvinylidene-fluoridetrifluoroethylene (PVDF-TrFE)/MXene nanofiber mats. The PVDF-TrFE nanofiber sensor has a response time of 15 ms and a recovery time of 20 ms and is capable of recording mechanical shocks and acoustic vibrations. The hydrogel sensor has a humidity response time of 50 ms. When the humidity suddenly increases from 30% RH to 90% RH, the change in conductivity is less than 10%, making it suitable for monitoring in humid environments such as bathrooms and outdoors [41].

### 3.2. Stability in Cycles

The zinc-ion pool-type battery sensor avoids electrode wear through a porous isolation layer, resolving the cyclic failure issue of conventional battery-type sensors. After 1200 pressure cycles at 48 kPa, its output attenuation is less than 3%, and the storage capacity is still above 90% after ten charge–discharge cycles [86]. For iontronic capacitive sensing, the sensing performance of the self-powered capacitive sensor can remain almost constant within the frequency range of 1 to 10 Hz [115,116].

The waterproof fabric-based triboelectric nanogenerator (TENG) maintains consistent operation after undergoing 1000 mechanical impacts (30 N), five washing cycles, and a 5-day water immersion test. This waterproof fabric TENG achieves a maximum raindrop energy density of 19.53 μWh/cm^2^ and a maximum wind energy density of 70 μWh/cm^2^ and can charge a 1 μF capacitor to 9 V within 30 s. When integrated with machine learning, the artificial intelligent self-powered TENG sensor achieves a 96.67% gait recognition accuracy and a 99.167% gesture recognition accuracy via 1D Convolutional Neural Network (1D CNN) [117].

### 3.3. Self-Powered Modes and Efficiency

Microbial biofilm sensors can continuously generate electricity for 380 h based on humidity gradients, with an open-circuit voltage fluctuation of less than 10% [63]. Zinc-ion battery-type sensors can be recharged to supplement energy, boasting a power density of 0.54 mV/cm^−2^ and an energy density of 392 μWh/cm^−3^ [86]. The tribo-ionic conduction synergy of gel-based TENGs enhances the energy conversion efficiency by three times compared with other TENGs [38,41]. As shown in Table 3, the quantitative parameters of energy conversion efficiency of different types of sensors are compared.

Based on the above discussion, we have compiled a comparison of the core performance indicators of different types of thermoelectric sensors, as shown in Table 4.

## 4. Typical Application Scenarios of Self-Powered Sensors

Self-powered sensors, with their flexibility and low power consumption, can currently be applied to a variety of typical scenarios such as wearable medical and health monitoring [118], intelligent robots [119] and electronic skin [42], human–machine interaction [120], environmental industrial monitoring intelligent security [121], and document management [122]. Specifically, these include the following:

### 4.1. Wearable Medical and Health Monitoring

Wearable scenarios place extremely high demands on the flexibility, biological stability, and long-term stability of sensors. The application scope in the health field ranges from power supplies for cardiovascular electronic devices to active endocardial monitoring [123]. TENG technology, thermoelectric, Seebeck transport, water-volt, and photovoltaic technologies are the mainstream choices. In the field of healthcare, self-powered sensors could be applied to the detection of medical physiological markers such as glucose, lactic acid, ethanol, thrombin, and acetylcholine. Gait recognition based on TENG smart socks (accuracy rate of 96.67%) can distinguish actions such as walking and jumping through 1D CNN algorithms [124].

**Figure 10:**
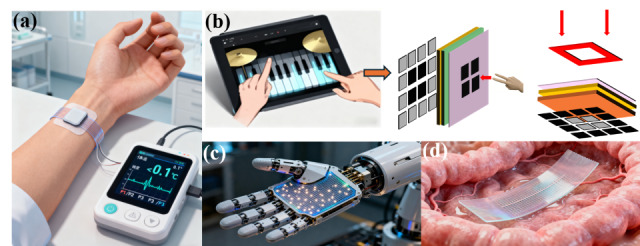
Typical application scenarios of self-powered sensors. (**a**) Application scenario of a self-powered wearable health monitoring sensor driven by human thermal energy; (**b**) schematic diagram of multi-point contact for “O” handwriting trajectory of flexible pressure sensor with color-tunable triboelectricity-induced electroluminescence; (**c**) schematic illustration of the sensor array integrated into a robotic palm for tactile sensing; (**d**) implantable sensors can track intestinal movement to diagnose diseases.

The temperature-pressure dual-peak sensor based on Bi-TE thermoelectric films and porous microcone elastomers takes Bi_2_Te_3_ and Sb_2_Te_3_ thermoelectric films as the temperature sensing core, collects human thermal energy through the Seebeck effect, and achieves a temperature sensitivity of 3.77 mV/K and a resolution of <0.1 K. The pressure sensing section adopts a porous microcone PDMS elastomer, achieving a high sensitivity of 37 kPa^−1^ and an ultra-low detection limit of 16 Pa. The sensor stabilizes the cold end temperature through a flexible heat sink design, which can be directly attached to human skin. It simultaneously monitors wrist pulse (identifying P wave, T wave, and D wave peaks, with a pulse rate measurement error of less than 2%) and body surface temperature (error of less than 1.1%). The response time is only 60 ms and the performance attenuation in the 3-month test is only 3.2%. As shown in Figure 10a, such a self-powered sensor can serve as a health detector based on human thermal energy.

### 4.2. Intelligent Robots and Human–Machine Interaction

Intelligent robots and human–machine interaction require sensors to have rapid response, spatial resolution, and multi-modal perception capabilities. Self-powered sensors achieve functions such as tactile mapping and gesture recognition through mechanisms like triboelectricity and piezoelectricity and require no additional power supply modules [125,126].

For precision assembly robots, based on solid–liquid friction, mechanical finger sensors can be divided into stages of touch, hold, and release. When grasping objects, the voltage variation clearly reflects the pressure change (0–10 N) and the positioning error is less than 1 mm [35]. Through array design, the hierarchical mode self-powered sensor has achieved multi-touch mapping with resolution of 500 μm on the robot’s palm.

Su et al. reported a new class of SP-VFPS enabling a color-tunable TIEL in response to mechanical pressure for the first time, comprising two parts, namely a porous red photoluminescence (PL) architecture and a TIEL component composed of luminescent and electrification layers [127]. The color-adjustable triboelectric electroluminescence (TIEL) visualization flexible pressure sensor is composed of a porous red PL structure and a green TIEL component. It is self-powered through the triboelectric effect, with a pressure detection limit as low as 10 kPa, a sensitivity of over 190 kPa^−1^, and a response time of less than 10 ms. After integration with the 4 × 4 TENG matrix, a hybrid sensing system is formed, which can achieve spatial pressure mapping with a resolution of 500 μm—when the robot grasps an object, the sensor visualizes the pressure distribution through color changes (adjusting the intensity ratio of green TIEL to red PL). In human–computer interaction, it can recognize handwritten trajectories and gesture strength, and transmit the information wirelessly via optical signals, thereby avoiding congestion in radio frequency communication. It is compatible with the touch skin of robots and touch control of intelligent terminals. After 20,000 cycles of testing, the luminous intensity has not decreased, and the stability is excellent [128]. As shown in Figure 10b, it is a schematic diagram of the handwritten trajectory contact of “O”. As shown in Figure 10c, it is the schematic illustration of the sensor array integrated into a robotic palm for tactile sensing.

### 4.3. Safety and Environmental Monitoring

By leveraging battery-integrated architecture and hydroelectric systems, self-powered sensors could achieve long-term, unsupervised monitoring thanks to their robust performance in sustained and autonomous operation [128,129], making them an ideal choice for applications such as industrial safety and ecological supervision. A trifuloroethylene (ECTFE) sensor based on TENG was proposed to monitor the marine environment [129,130].

The waterproof fabric multi-functional triboelectric nanogenerator (WPF-MTENG) integrates rainwater/wind energy collection and environmental anomaly monitoring in response to the demand for distributed deployment with resistance to harsh environments in safety and environmental monitoring. The key designs include the following: a multi-layer fabric structure (with a conductive fabric-rubber film layer encapsulated by EVA film at the bottom and a mesh fabric-conductive fabric composite layer at the top), achieving energy conversion through a contact separation mode. The porous structure of the EVA film (without penetrating holes) ensures water resistance, and there is no performance degradation after five washes and 5 days of soaking. The surface of the rubber membrane is roughened by molding with 425 µm SiC paper and combined with mesh fabric to form an effective air gap, maximizing the triboelectric output (output voltage 850 V/m^2^ at a rainfall of 125 mL/s and 900 V/m^2^ at a wind speed of 15.4 m/s) [33].

### 4.4. Biomedical and Implantable Uses

Implantable sensors need to meet the requirements of biocompatibility, degradability, and low invasiveness. Self-powered sensors achieve energy self-sufficiency through energy within the organism such as body fluid flow and tissue movement or biocompatible materials and are suitable for monitoring internal pressure and physiological signals. Photovoltaic sensors based on microbial biofilms have strong biocompatibility and can be implanted in mice to measure blood pressure (50–150 mmHg) [63]. The system ran constantly for more than 30 days, with the output voltage remaining constant between 0.2 and 0.4 V. 

Jia et al. [87] reported a gelatin-based battery type pressure sensor. The gelatin-chitosan composite hydrogel battery-type pressure sensor uses a gelatin-chitosan composite film as the sensing layer, VO_2_-coated stainless-steel mesh and zinc sheet as electrodes, and PDMS as the isolation layer. The self-powered mechanism is derived from the REDOX reaction of zinc-ion batteries. The sensor features a high sensitivity of 37.9 mV/kPa, a detection range of up to 524 kPa, response and recovery times of 177 ms and 193 ms, respectively, and can be completely degraded in moist soil within 10 days, with excellent biocompatibility [87]. In implantable applications, this sensor can be implanted on the surface of human organs (such as the gastrointestinal tract and cardiovascular system) to monitor dynamic pressure changes or used for postoperative tissue pressure monitoring, avoiding the risk of external power supply wiring. As shown in Figure 10d, it is an implantable sensor for tracking intestinal movement. Its stable cycle exceeds 10,000 s, with a voltage drop of only 0.07 V, meeting the long-term in vivo monitoring requirements. Meanwhile, its degradable property avoids secondary surgical removal and reduces medical risks [131].

## 5. Present Challenges and Future Prospects for Self-Powered Sensors

### 5.1. Present Challenges

Based on the research progress of the aforementioned types of sensors, the current challenges faced by self-powered sensors can be summarized as follows: energy supply stability, trade-offs in sensing performance, inherent defects in materials and structures, and mismatches between data processing and practical application scenarios.

The issue of energy supply stability is mainly reflected in the reliance of traditional self-powered sensors on a single energy source. Early triboelectric sensors could only harvest one type of energy, such as raindrop energy, wind energy, or human motion energy, failing to adapt to complex environments with coexisting multiple energy sources. Although attempts have been made to integrate photovoltaics with f-TENG to achieve “light energy + mechanical energy” dual-source harvesting, the process is complex and poorly compatible, making mass production difficult. Furthermore, triboelectric sensors are prone to weakened triboelectric effects due to water molecules; in wearable scenarios, human perspiration or rainy environments can reduce their power generation capacity [36,132].

The trade-offs among multiple objectives in sensing performance present a complex and multifaceted challenge. Self-powered sensors need to simultaneously meet requirements such as “high sensitivity, wide detection range, fast response, and static detection,” but these indicators are often contradictory, making it challenging to balance all needs. For example, there is an inverse trade-off between sensitivity and detection range: optimizing microstructures can improve sensitivity in the low-pressure region, but the high-pressure region is prone to reduced linearity due to “contact saturation,” limiting the detection range (the sensitivity of most sensors in the high-pressure region is only 1–2 mV/kPa) [127]; although porous structures can expand the detection range to hundreds of kPa, the sensitivity is significantly reduced, failing to balance “micro-force detection” and “large-pressure response” [118].

The problem of inherent defects in materials and structures mainly stems from the dependence of sensor performance on material properties and structural design, but there are still various limitations in current material selection and structural optimization. For instance, wearable scenarios require electrodes to have both flexibility and high conductivity, but traditional metal foil electrodes are prone to fracture under bending or stretching [50]; battery-type sensors often use hydrogel electrolytes, but the gel is prone to adhesion with electrodes or separators, damaging the “pressure-impedance” conversion mechanism. At room temperature, hydrogels are susceptible to water loss, leading to a decrease in ionic conductivity, which requires additional packaging. However, packaging can reduce the flexibility of the device [41,133].

With the integration of self-powered sensors and artificial intelligence (AI), the contradiction between “data quality” and “algorithm adaptation” has become prominent. For example, triboelectric sensors rely on mechanical stimulation to generate electrical signals, but the randomness of environmental energy results in “fragmented” data. The irregularity of human motion causes large fluctuations in the output signals of triboelectric sensors, with small single-sample data volume, making it difficult to meet the demand for “large-scale training data” by AI algorithms. Traditional AI algorithms are designed based on “complete data,” but self-powered sensors may generate low precision data when energy is insufficient. Existing algorithms cannot effectively recover incomplete information, and new machine learning methods need to be developed, but related research is still in its infancy [117,134].

Although self-powered sensors can meet the complex needs of wearable, medical, industrial, and other scenarios, they still have obvious shortcomings in biocompatibility, long-term stability, and environmental adaptability. Some materials (such as PDMS and metal electrodes) irritate the skin; for example, although microbial biofilm sensors are fixed with medical PU tape, long-term wear may cause skin itching [6]. Meanwhile, microbial biofilm sensors require packaging to maintain humidity, but packaging materials are prone to rupture due to mechanical deformation. In industrial scenarios, dust and chemical reagents can contaminate electrodes, leading to device failure, and existing packaging processes struggle to balance protection and flexibility. Additionally, there is a contradiction between flexibility and breathability: although packaged sensors are moisture-proof, their poor breathability prevents the discharge of human perspiration, significantly affecting the wearing experience.

### 5.2. Future Prospects

In response to the above challenges, in combination with the technical directions in the literature, self-powered sensors will develop in the following four major directions: material innovation, structural optimization, intelligent integration, and scene adaptation.

Material innovation should develop towards multi-functionality and intelligence: Intelligent responsive materials can be developed in combination with stimuli-responsive materials, such as temperature-sensitive hydrogels, which soften at the wearer’s body temperature (37 °C) to fit the skin well and harden to maintain shape at ambient temperature (25 °C), enhancing wearing comfort and stability [46,135].

Structural optimization is achieved through miniaturization and integration: By using 3D printing technology or introducing patterned microstructure to fabricate complex-structured sensors [136], such as microfluidic-sensing integrated devices, the channel diameter can be reduced to micro scale, enabling the synchronous collection and detection of trace body fluids. Integrate self-powered sensors with microprocessors, memory, and wireless modules to develop zero-power intelligent nodes, such as wearable health monitoring modules integrated with TENG, which have a volume of less than 1 cm^3^ and a weight of less than 5 g and can work continuously for 6 months without charging [137].

The integration of algorithms through AI empowerment and edge computing: Deep learning algorithms can enhance the accuracy of signal recognition, and federated learning technology can address the privacy issues of multi-sensor data, making it suitable for medical and health scenarios. Integrate edge computing chips at the sensor end to achieve real-time data processing, reduce data transmission volume, and lower power consumption. For example, for self-powered sensors integrated with RISC-V architecture chips, the data processing latency is less than 1 ms and the power consumption is less than 5μW [138].

Achieve scene adaptation through cross-scene customization: For self-powered devices based on triboelectric charge transfer and electrostatic induction (TENG), as well as the piezoelectric effect, the fundamental mechanism is to convert external mechanical stimuli, such as pressure, vibration, or motion into electrical energy [139,140]. The five main forms of energy—light, heat, mechanical, electrical, and magnetic—are essentially interconnected and can undergo various coupling and conversion. Among them, electricity and magnetism correspond closely and can achieve efficient interaction through electromagnetic induction, a mechanism that forms the basis of electromagnetic motors and electric generators.

### 5.3. A Map of Research Achievements in Recent Years

**Figure 11:**
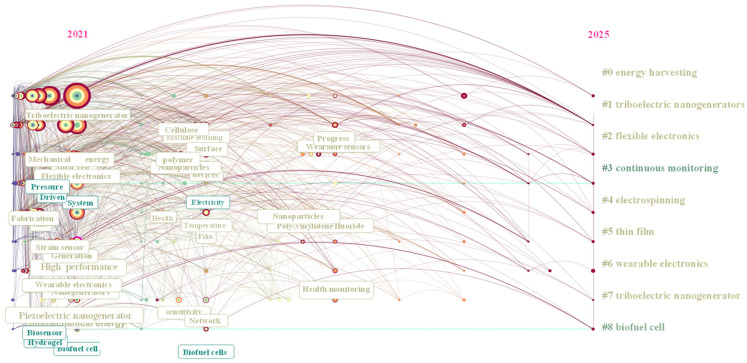
The development of self-powered sensors during 2021 to 2025.

The retrieval method using CitesSpace 6.4R1 with data from Web of Science is shown in Table 5. The time period from January 2021 to December 2025 was selected, and network nodes were chosen as keywords to obtain the keyword graph related to the research field of self-powered sensors, as shown in Figure 11. The *X*-axis of this keyword time series graph represents the years, covering the time interval from 2021 to 2025. The *Y*-axis represents the keywords related to the research field of self-powered sensors, presenting the correlation and migration of different topics in the time dimension through nodes and connections. The network has 288 nodes and 1532 edges, with a network density of 0.0371. The low density suggests that the main topics are focused while the keywords in the area are rather scattered. The modularity Q is 0.3699, implying distinct theme groupings within the area. With a mean silhouette score S of 0.6898, it denotes strong cluster differentiation and good internal consistency within clusters. The size of the nodes of keywords indicates how frequently they occur, with larger nodes denoting higher frequency.

From the perspective of timeline evolution, as reflected by node color and spatial distribution, together with clustering relationships characterized by link density, the development of this field can be mainly divided into three stages. The early stage is represented by cool-colored nodes (such as blue and purple), with research focused on basic materials and preparation techniques, such as electrospinning (#4) and thin-film processes (#5). These foundational material technologies laid an important basis for flexible electronics and energy-harvesting devices. The intermediate stage, characterized by green and yellow nodes, emphasizes key device breakthroughs, including triboelectric nanogenerators (#1) and biofuel cells (#8), with attention to principle validation and performance optimization. In the recent stage, research characterized by red and orange nodes places more emphasis on integrated applications and system-level innovations, such as flexible electronics (#2), continuous monitoring systems (#3), and wearable electronics (#6). This stage demonstrates a deep integration of flexible materials, energy harvesting, and continuous sensing, reflecting a transition from technological development to practical application.

Overall, the cross-disciplinary nature of the technology is reflected in the close integration of energy harvesting (#0) with key device technologies (#1, #8), while flexible electronics (#2), wearable electronics (#6), and continuous monitoring (#3) together form a coherent material–device–application closed loop. In recent years, warmer-colored nodes such as wearable electronics (#6) and continuous monitoring (#3) have shown larger node sizes and denser connections, indicating that human-centered health and everyday life scenarios are becoming the core direction for technology deployment. Technology iteration is reflected in the gradual shift in energy harvesting from traditional batteries toward environmentally friendly technologies (such as TENG and biofuel cells), and device manufacturing is also accelerating from rigid structures toward flexible and wearable forms (such as electrospinning and film technologies).

The cluster labels on the right correlate to the node color; various colors denote distinct groupings of study themes. Node locations over the timeline are as follows: the horizontal axis, which runs from 2021 to 2025, depicts the development of research hotspots over time, with early hotspots on the left and new routes on the right. Around 2021, the important key themes in the fundamental technology and materials layer included the following: with their massive and dense nodes, triboelectric nanogenerators (#1) and piezoelectric nanogenerators (#5) are key components of nanogenerator technology, which represents fundamental advancements in energy harvesting technology. Flexible and miniaturized materials, such as flexible substrates for wearable devices, are the focus of supporting technologies such as thin film (#5), electrospinning (#4), and flexible electronics (#2).

Energy harvesting (#0) and bioenergy of biofuel cells (#8) are the two major energy acquisition directions. While biofuel cells convert biochemical energy to electrical energy, triboelectric/piezoelectric nanogenerators convert mechanical energy to electrical energy.

Typical examples of application and scenario layer in the evolving direction towards 2025 include wearable electronics (#6), continuous monitoring (#3), and health monitoring, illustrating the evolution of research toward device integration, performance enhancement, and system-level applications in nodes such as mechanical energy, pressure sensor, fabrication, and system. Technical routine is developed follows: from “basic nanogeneration technologies of triboelectric/piezoelectric to flexible material fabrication electrospinning/films to wearable device integration to health monitoring and other application scenarios,” forming a complete technology chain. With a hotspot evolution showing an industry-oriented strategy of “from technology to product”, the emphasis shifted from “materials and device principles” about 2021 to “system applications and scenario implementation” by 2025. Multidisciplinary cooperation is presented as follows: emerging areas like “intelligent wearable energy systems” and “non-invasive health monitoring” are the result of the close intersections between energy harvesting technologies like nanogenerators and biofuel cells and industries like flexible electronics, wearable technology, and healthcare. The field’s evolutionary route from “basic research to technological breakthroughs to application expansion” is clearly shown in this diagram, which serves as a visual aid for comprehending research boundaries and formulating scientific research directions.

**Figure 12:**
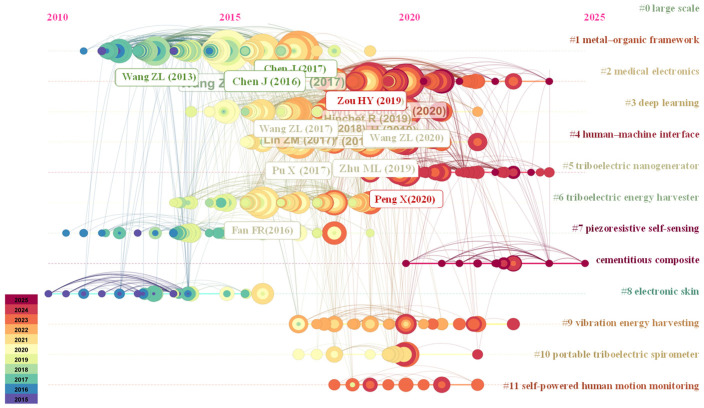
The development of the last decade regarding self-powered sensors.

To further study the evolution of self-powered sensors in the last decade, Citespace 6.4 R1 was continued to be used. The search steps are shown in Table 5, and the time series diagram of the cited literature is shown in Figure 12. The horizontal axis of the time series graph of the cited literature represents the publication time, covering the time interval from 2015 to 2025. The vertical axis represents the subject of the literature. The size of a node represents the number of cited documents; most cited references are concentrated between 2015 and 2023, suggesting significant research achievements in the field of self-powered sensors during this period.

Inter-cluster associations and cross-innovation are reflected through the thickness and color of the connecting lines (corresponding to different years), representing the co-citation strength between fields. The main cross-nodes include the following: (a) in the energy-sensing-medical direction, triboelectric nanogenerators (#5, #6, #9) are closely coupled with self-powered monitoring (#11), electronic skin (#8), and spirometers (#10), driving the development of passive sensing systems that operate without external power and rely on human or environmental energy; (b) in the materials-smart systems direction, functional materials such as metal–organic frameworks (#1) and piezoresistive cement (#7) serve as key supports, facilitating the miniaturization and intelligence of medical electronics (#2) and human–machine interaction (#4) devices; (c) in the AI-energy optimization direction, deep learning (#3) provides algorithmic support for predicting energy harvesting efficiency and processing sensor data, reflecting the trend of cross-innovation where materials and energy technologies are integrating intelligently. Among emerging materials, cement-based thermoelectric materials represent a promising option for developing sustainable structures [91].

## 6. Conclusions

This review confirms that the six core technical paths of self-powered sensors—including triboelectric charge transfer and electrostatic induction, thermoelectric Seebeck transport, hydrovoltaic interfacial streaming potentials, piezoelectric constitutive relations, battery integration mechanism, and photovoltaic effect—have achieved significant breakthroughs in key performance indicators through material innovation and structural optimization, and the differentiated advantages of each technology are suitable for different scenario requirements. Although self-powered flexible sensors have made significant progress, they still face core challenges such as insufficient stability of energy supply, trade-offs in performance parameters, material and structural defects, bottlenecks in integrated manufacturing, and poor adaptability in data processing. In response to the above challenges, based on experience, this study proposes the following four specific development directions: in-depth material innovation, structural optimization, intelligent integration, and scene adaptation.

This review, through systematic sorting and critical analysis, clearly indicates that self-powered flexible sensors have moved from the stage of basic material exploration to the stage of scenario-based application. Their integrated capability of energy harvesting and signal perception provides a new paradigm for fields such as flexible electronics, intelligent healthcare, and the Internet of Things. In the future, it is necessary to further focus on the following three major directions: multi-source energy coupling mechanism, material interface regulation, as well as AI and low-precision data adaptation algorithms, to promote the technology from laboratory verification to industrial mass production, and ultimately achieve the goal of highly integrated scenario-based Internet of Things applications with zero power consumption. It is crucial that we provide technical support for the upgrade of flexible industries and the security of people’s livelihood and health.

## Figures and Tables

**Table 1:** Performance comparison of different types of thermoelectric sensors under different test conditions.

Sensor Type	Performance Value	Test Conditions
Thermoelectric sensing (Bi_2_Te_3_ thin film)	1. Room temperature conductivity: Bi_2_Te_3_ is 5.1 × 10^4^ S m^−1^. 2. Power factor: Sb_2_Te_3_ is twice that of Bi_2_Te_3_.	1. Thermoelectric performance test: Measured by ZEM-3 instrument. 2. Temperature range: within 100 °C 3. Film preparation: sputtering at 350 °C and 2 Pa for 4 h.
Thermoelectric sensing (TEG integration)	1. 0.1 K temperature difference output voltage 0.36 mV. 2. Periodic output of 8.1 mV at 33 °C hot side/31 °C cold side. 3. The temperature measurement deviation of the hot cup is ≤ 1.1%.	1. Temperature test: industrial refrigeration chiller temperature control, temperature difference 0.1 to 100 K. 2. Pressure coupling test: apply a load of 16 to 5800 Pa to the stepping motor at a frequency of 0.2 to 2 Hz.

**Table 2:** Comparison of thermoelectric/piezoelectric sensing benchmarks.

Sensor Type	Coefficient	Temperature Resolution	Thermal Range	Cyclic Stability	Uncertainty (±)	Source of Literature
Thermoelectric sensing (Bi_2_Te_3_ thin film)	Bi_2_Te_3_: −180 μV/K; Sb_2_Te_3_: 240 μV/K	<0.1 K	0~100 °C	It declined by 3.2% in three months	Seebeck coefficient ±5 μV/K; temperature resolution ± 0.01 K	[110]
Thermoelectric sensing (TEG integration)	3.77 mV/K	<0.1 K	23.8~35.8 °C	**_**	Sensitivity: ±0.05 mV/K; temperature resolution ±0.05 K	[110]
Piezoelectric sensing (GaN nanowires)	**_**	**_**	25 ± 5 °C	Mechanical cycle > 5000 times	**_**	[113]
Piezoelectric sensing (BOPET film)	**_**	**_**	Room temperature to 245 °C	80 pressure cycle outputs	Output voltage ± 2 V	[114]

**Table 3:** Comparison of quantitative parameters for energy conversion efficiency of different types of sensors.

Sensor Type	Core Materials/Structure	Quantitative Parameters Related to Energy Conversion
Triboelectric nano- generator (TENG) [33]	WPF-MTENG	Rainfall: maximum power density 0.35–19.53 μW/m^2^; Wind energy: maximum power density 30 to 70 μW/m^2^.
Hydrovoltaic effect-based sensor [63]	mBio-HPS	Peak power density ≈ 1500 μW/m^2^ (150 nW/cm^2^).
Battery-integrated sensor [86]	RZIB-FPS	Energy density: 392 µWh/cm^2^; Power density: 5.4 W/m^2^ (0.54 mW/cm^2^).
Gel-based TENG (aerogel type) [39]	CCA-TENG	The power density is 1237 mW/m^2^; After 64,800 cycles, the performance remains at 91.04%.
Gel-based TENG (organic hydrogel type) [39]	MX-GO/CNF/ SA/PVA	Energy density: 392 µWh/cm^2^; Power density: 5.4 W/m^2^ (0.54 mW/cm^2^).
Gel-based TENG (ionic gel type) [39]	IG 70–10%	The maximum power density is 157.1 mW/m^2^; The ionic conductivity is 2.18 mS/cm.

**Table 4:** The parameters for typical self-power sensors.

Mechanism Mode	Materials	Sensing Type	Range	Sensitivity	Response Time	Refs.
Gel-based TENGs	Hydrogel	TENG	1.3 Pa~6.83 MPa	0.59 μA/kPa	10 ms~112.5 μs	[41]
mBio-HPS	G.S	Hydrovoltaic effect-based sensor	16 Pa~25 kPa	2241.49 kPa^−1^	112.5 μs	[63]
RZIB-FPS	PVA-GO	Battery-integrated sensor	126–330 kPa	1.18 mVkPa^−1^	96.0 ms	[86]
Temperature/pressure sensor	Bi_2_Te_3_	Thermoelectric-based sensor	≤100 °C	3.77 mV·K^−1^	24 ms	[99]
Sensor based on piezoelectric	PVDF-TrFE	Piezoelectric pressure sensor	18~79 N	1.29 mV/(μm·N)	16~56 ms	[71]

**Table 5:** Retrieval process for obtaining statistics of self-powered sensors.

Search Type	Search Method
Database	Web of science
Theme	Self-powered sensor
Number of studies	1000
Boolean retrieval expression	And
Time range (Figure 11)	January 2021–December 2025
Time range (Figure 12)	January 2015–December 2025
Node (Figure 11)	Keywords
Node (Figure 12)	Cited References

## Data Availability

No new data were created or analyzed in this study.
